# Beautiful Snow (Poetry)

**Published:** 1881-01

**Authors:** 


### BEAUTIFUL SNOW.

Oh, the snow, the beautiful snow;
Filling the sky and the earth below;
Over the housetops, over the street;
Over the heads of the people you meet;
Dancing,
Flirting,
Skimming along.
Beautiful snow! It can do no wrong—
Flying to kiss a fair lady's cheek,
Clinging to lips in frolicsome freak.
Beautiful snow from the heavens above!
Pure as an angel, but fickle as love!

Oh, the snow, the beautiful snow!
How the flakes gather and laugh as they go!
Whirling about in their maddening fun,
They play in their glee with everyone;
Chasing,
Laughing,
Hurrying by,
It lights on the face and sparkles the eye;
And even the dogs, with a bark and a bound,
Snap at the crystals that eddy around.
The town is alive and its heart in a glow,
To welcome the coming of beautiful snow.

How the wild crowd goes swaying along,
Hailing each other with humor and song!
How the gay sledges like meteors flash by,
Bright for a moment, then lost to the eye!
Ringing,
Swinging,
Dashing they go,
Over the crust of the beautiful snow.
Snow so pure, when it falls from the sky,
To be trampled in mud by the crowd rushing by;
To be trampled and tracked by the thousands of feet,
Till it blends with the filth in the horrible street.

Once I was pure as the snow; but I fell—
Fell, like the snow flakes, from heaven to hell;
Fell, to be trampled as filth in the street;
Fell, to be scoffed, to be spit on and beat;
Pleading,
Cursing,
Dreading to die,
Selling my soul to whoever would buy;
Dealing in shame for a morsel of bread;
Hating the living and fearing the dead.
Merciful God! have I fallen so low?
And yet I was once like the beautiful snow.

Once I was fair like the beautiful snow,
With an eye like its crystal, a heart like its glow;
Once I was loved for my innocent grace,
Flattered and sought for the charms of my face.
Father,
Mother,
Sisters and all,
God and myself I have lost by my fall.
And the veriest wretch that go shivering by
Will make a wide swoop lest I wander too nigh;
For, of all that is on or about me, I know
There is nothing that's pure but the beautiful snow.

How strange it should be that the beautiful snow
Should fall on a sinner with nowhere to go!
How strange it should be if, ere night comes again,
The snow and the ice strike my desperate brain!
Fainting,
Freezing,
Dying alone—
Too wicked for prayer, too weak for a moan
To be heard in the crash of the crazy town,
Gone mad in the joy of the snow coming coming down,
I should lie and should die in my terrible woe,
With a bed and a shroud of the beautifu snow!

Helpless and foul as the trampled snow,
Sinner, despair not! Christ stoopeth low
To rescue the soul that is lost in its sin,
And raise it to life and enjoyment again.
Groaning,
Bleeding,
Dying for thee,
The Crucified hung on the accursed tree.
His accents of mercy fall soft on thine ear.
Is there mercy for me? Will He heed my prayer?
Oh, God! in the stream that for sinners did flow,
Wash me, and I shall be whiter than snow.

